# A Phase 2 study of nivolumab in combination with modified FOLFIRINOX for metastatic pancreatic cancer

**DOI:** 10.1038/s44276-023-00028-4

**Published:** 2024-01-23

**Authors:** Chigusa Morizane, Makoto Ueno, Masafumi Ikeda, Kentaro Sudo, Yoshinori Hirashima, Masataka Kuroda, Shinji Ueno, Takuji Okusaka, Junji Furuse

**Affiliations:** 1 Department of Hepatobiliary and Pancreatic Oncology, National Cancer Center Hospital, Tokyo, Japan; 2 Department of Gastroenterology, Hepatobiliary and Pancreatic Medical Oncology Division, Kanagawa Cancer Center, Yokohama, Japan; 3 Department of Hepatobiliary and Pancreatic Oncology, National Cancer Center Hospital East, Kashiwa, Japan; 4 Department of Gastroenterology, Chiba Cancer Center, Chiba, Japan; 5 Clinical Development Headquarters, Ono Pharmaceutical Co., Ltd., Osaka, Japan; 6 Department of Medical Oncology, Kyorin University Faculty of Medicine, Mitaka, Japan; 7 Present Address: Kanagawa Cancer Center, Yokohama, Japan

## Abstract

**Background:**

Nivolumab with modified FOLFIRINOX (mFOLFIRINOX) may have additive antitumour effects while minimising chemotherapy cytotoxicity. We assessed the efficacy and safety of nivolumab+mFOLFIRINOX in metastatic pancreatic cancer.

**Methods:**

Thirty-one treatment-naïve patients aged ≥20 years with metastatic unresectable/recurrent pancreatic cancer (≥1 measurable lesion per Response Evaluation Criteria in Solid Tumours version 1.1) and Eastern Cooperative Oncology Group 0/1 score and life expectancy ≥90 days received nivolumab (480 mg, every 4 weeks) plus mFOLFIRINOX. The primary endpoint was objective response rate (ORR). Secondary endpoints included overall survival (OS), progression-free survival (PFS) and safety.

**Results:**

At the median follow-up of 13.4 months, the ORR was 32.3% (complete response 0%; partial response 32.3%) and the median duration of response was 7.4 (range: 3.5–21.9) months; the primary endpoint was not met. Median OS and PFS were 13.4 (95% confidence interval [CI]: 10.6–16.6) months and 7.4 (95% CI: 3.9–9.2) months, respectively. The 1-year survival rate was 54.8% (95% CI: 36.0%–70.3%). Drug-related serious adverse events were reported in 29.0% of the patients; 3.2% drug-related adverse events led to discontinuation, and none led to death within 30-day safety window.

**Conclusion:**

Nivolumab+mFOLFIRINOX was tolerable in patients with metastatic pancreatic cancer. ORR and survival were comparable to previously reported data. (JapicCTI-184230)

## Background

Pancreatic cancer ranks among the top five gastrointestinal cancers [1]. The majority of pancreatic cancers are adenocarcinomas, accounting for approximately 85%–95% of all pancreatic tumours [2]. Unfortunately, owing to lack or non-specificity of symptoms in early stages, most patients with pancreatic cancer present clinically at an advanced stage and have grim prognoses [3]. It is one of the most lethal cancers despite the advances in cancer therapeutics. In fact, pancreatic cancer is predicted to become the third leading cause of death from cancer in Europe by 2025 [4] and second leading cause of death from cancer in the United States by 2030 [5].

Both FOLFIRINOX and gemcitabine + nab-paclitaxel regimens are considered standard of care worldwide and also in Japan for unresectable metastatic pancreatic cancer [6–9]. However, these regimens have not greatly influenced overall survival (OS; median survival being <1 year) in these patients, with no substantial improvement in survival in the last 10 years [10, 11], despite the high costs as well as toxicity [10, 12].

Recently, in a randomised, open-label, Phase 3 trial, first-line NALIRIFOX (liposomal irinotecan, fluorouracil, leucovorin and oxaliplatin) demonstrated significant improvement in OS and progression-free survival (PFS) over gemcitabine + nab-paclitaxel in treatment-naïve patients with metastatic pancreatic ductal adenocarcinoma [13]. However, the median OS was still <1 year (11.1 months) in NALIRIFOX arm, suggesting a limited impact on the therapeutic landscape.

Nivolumab, a monoclonal antibody, binds to programmed death-1 receptor (PD-1), preventing binding of ligands to PD-1. Thus, nivolumab prevents downstream immune suppression and elicits an immune response [14]. It has shown efficacy in multiple cancer types both as monotherapy and in combination with other therapies [15–19]. Understanding of the cancer–immunity cycle is useful in elucidating how the combined use of nivolumab and FOLFIRINOX could have a synergistic antitumour effect owing to their different yet complementary mechanisms of action. The development of cancer immunity is a self-propagating and self-amplifying virtuous cycle that includes cancer cell antigen release, T cell activation, infiltration of T cells into tumours, and the killing of cancer cells [20]. Chemotherapy has been described to not only directly inhibit the proliferation of cancer cells but also restore the immune surveillance mechanism for cancer cells and thus provide better immune environment for subsequent immunotherapy [21–23]. As chemotherapy with drugs such as oxaliplatin induces cancer cell death, exposure of the tumour antigen to antigen-presenting cells increases, thereby potentiating the cancer elimination mechanism [24, 25]. 5-fluorouracil and oxaliplatin have been reported to eliminate myeloid-derived suppressor cells and other immune-related cells [26, 27]. At the same time, irinotecan enhances the effect of T cell activation caused by anti-programmed death-ligand 1 (PD-L1) treatment; irinotecan leads to depletion of regulatory T cells, with restoration of the cancer immune surveillance mechanism [28]. Thus, chemotherapy can expand the response of the cancer–immunity cycle improving the efficacy of nivolumab.

Therefore, we evaluated the combined antitumour efficacy and safety of nivolumab, an immune checkpoint inhibitor, and a standard chemotherapeutic regimen of modified FOLFIRINOX (mFOLFIRINOX) vs. current standard of care in patients with chemotherapy-naïve metastatic pancreatic carcinoma.

## Methods

### Study design and treatment

This was a multicentre, open-label, Phase 2 study (JapicCTI-184230) conducted at five centres; the first and the last patients were enroled on 10 January 2019, and 09 July 2019, respectively. The study (JapicCTI-184230) was conducted in compliance with the protocol, the Declaration of Helsinki, the International Council for Harmonisation of Technical Requirements for Pharmaceuticals for Human Use Good Clinical Practice Guidelines and other applicable laws and regulations. It was also approved by the Institutional Review Board of each study site. All participants gave their written informed consent for study participation.

### Patients

Patients aged ≥20 years with life expectancy ≥90 days at the time of enrolment were eligible for inclusion if they had histologically or cytologically diagnosed pancreatic adenocarcinoma with distant metastases without prior treatment for pancreatic cancer (except surgical resection) and ≥1 measurable lesion as per Response Evaluation Criteria in Solid Tumours version 1.1 (RECIST v1.1) guideline at imaging within 14 days before enrolment. Additional inclusion criteria were Eastern Cooperative Oncology Group (ECOG) performance status score of 0 or 1 [29]; adequate haemogram (neutrophil count ≥2000/mm^3^; platelet count ≥100,000/mm^3^; haemoglobin ≥9.0 g/dL) without receiving blood transfusion or a granulocyte colony stimulating factor within the past 14 days; adequate liver function (aspartate aminotransferase and alanine aminotransferase ≤100 U/L; total bilirubin ≤1.2 mg/dL); and adequate renal function (creatinine ≤1.2 mg/dL). Each patient voluntarily provided written consent.

Key exclusion criteria were current or past history of severe hypersensitivity reactions to antibody drugs; contraindicated use of FOLFIRINOX drugs; UDP-glucuronosyltransferase 1A1 (UGT1A1) homozygous (UGT1A1*6/*6, UGT1A1*28/*28) or double heterozygous (UGT1A1*6/*28) genotype; concurrent, chronic or recurrent autoimmune disease (Supplementary Material); multiple primary cancers; any symptomatic metastatic lesion in the brain or meninges requiring treatment; peripheral motor or sensory neuropathy; clinically relevant diarrhoea, diverticulitis or gastrointestinal ulcer disease; prior nivolumab or T cell suppression therapies; and vaccinations within 28 days before enrolment.

### Treatment

Treatment-naïve patients with metastatic unresectable/recurrent pancreatic cancer received nivolumab (480 mg, infused intravenously [IV] over 30 min every 4 weeks) plus mFOLFIRINOX. The mFOLFIRINOX regimen included oxaliplatin 85 mg/m² infused IV over 2 h, followed by levofolinate 200 mg/m^2^ infused IV over 2 h. Irinotecan 150 mg/m^2^ was infused IV over 1.5 h, starting 30 min after the start of levofolinate infusion. After the end of levofolinate infusion, fluorouracil 2400 mg/m^2^ was infused IV over 46 h. The treatment cycle of mFOLFIRINOX was repeated every 2 weeks.

No dose modification of nivolumab was permitted. If nivolumab and the mFOLFIRINOX regimen were given on the same day, nivolumab was administered first, and mFOLFIRINOX administration was started at least 30 min after the end of nivolumab infusion.

Treatment was repeated until the patient met any of the predetermined discontinuation criteria, which included intolerable adverse events (AEs), progressive disease as assessed by the investigator or sub-investigator (RECIST v1.1), worsened clinical symptoms judged to be due to disease progression and continuation of intervention judged to be inappropriate by the investigator or sub-investigator from efficacy or safety viewpoints.

### Assessments

At the beginning of a treatment cycle, the patients were evaluated based on medical history, complete physical examination by a physician, ECOG performance status and laboratory tests (haemogram, blood chemistry, qualitative urinalysis). Imaging was performed every 2 cycles (8 weeks). Patients were followed at 3-month intervals until death.

During the screening period, tumour tissue samples for analysis of PD-L1 expression status and tumour mutation burden (TMB; number of genetic mutations in tumour tissue) were collected. Tumour tissue samples were stained for PD-L1 and assessed by a pathologist at the central laboratory. PD-L1 expression was analysed with the pharmDx 28–8 assay (Dako, Carpinteria, CA, USA). The combined positive score (CPS) is the number of PD-L1 staining cells (tumour cells, lymphocytes, macrophages) divided by the total number of viable tumour cells, multiplied by 100. TMB was assessed using the Foundation Medicine’s solid tumour assay (Foundation Medicine, Cambridge, MA, USA).

### Endpoints

Progression was assessed according to RECIST v 1.1. The primary efficacy endpoint was the objective response rate (ORR) by central assessment and secondary efficacy endpoints included maximum percentage change in tumour diameter from baseline, OS, PFS by central assessment, duration of response, time to response, best overall response, disease control rate, percentage change and maximum percentage change in the sum of diameters of target lesions. Safety endpoints were AEs, clinical laboratory test results (haematology, blood chemistry, qualitative urinalysis, immunology tests, hormone tests) and vital signs (systolic and diastolic blood pressure, pulse rate, body temperature).

### Statistical analysis

All analyses were performed in the full analysis set. For each quantitative variable, the mean (standard deviation [SD]) was reported. For each qualitative variable, the number (%) was reported. The relative dose intensity was calculated as described in the Supplementary Material. For primary endpoints, 90% confidence intervals (CIs) were estimated using the Clopper-Pearson method. For secondary endpoints, the Kaplan–Meier method was used for time-to-event analysis. Specifically, for OS and PFS, Kaplan–Meier curves were prepared. The Kaplan–Meier method was used to calculate the median (95% CI) and the OS and PFS rates (95% CI) at 6, 12, 18 and 24 months. Exploratory analyses included efficacy assessments by PD-L1 status and by TMB.

For this study, assuming α = 0.10, a power of 80%, a threshold ORR of 31.6% (as per ACCORD 11 trial results [30]) and an expected ORR of 56.0% (based on clinical meaningfulness expectation), the minimum sample size required for the lower limit of the 90% CI of the ORR by the Clopper-Pearson method to exceed the threshold ORR was estimated to be 30 patients.

## Results

From five study sites, 31 patients were included in the study and in both the safety analysis and full analysis sets (Table 1).

**Table 1:** Baseline demographics and clinical characteristics.

Characteristics	Nivolumab + mFOLFIRINOX (*N* = 31)
Sex, male, *n* (%)	18 (58.1)
Age, median (range), years	59.0 (39–75)
ECOG performance status, *n* (%)	
0	21 (67.7)
1	10 (32.3)
Primary tumour site, *n* (%)	
Head	9 (29.0)
Body	14 (45.2)
Tail	11 (35.5)
Number of organs with metastases, *n* (%)	
1	20 (64.5)
≥2	11 (35.5)
Site of metastasis, *n* (%)	
Liver	20 (64.5)
Lung	5 (16.1)
Lymph node	14 (45.2)
Peritoneum	6 (19.4)
Pleura	1 (3.2)
CA19-9 (U/mL)^a^	
Mean (SD)	8125.5 (23,883.9)
Median [Minimum–Maximum]	1000.0 [1–113,900]
Any biliary drainage, *n* (%)	
No	24 (77.4)
Yes	7 (22.6)
PD-L1 (CPS), *n* (%)	
<1	23 (74.2)
≥1	7 (22.6)
Not quantifiable	1 (3.2)
Tumour mutation burden: (Muts/Mbp), *n* (%)	
<5	20 (64.5)
≥5	4 (12.9)
Missing	7 (22.6)

*CA* carbohydrate antigen, *CPS* combined positive score, *ECOG* Eastern Cooperative Oncology Group, *mFOLFIRINOX* modified FOLFIRINOX, *PD-L1* programmed death-ligand 1, *SD* standard deviation.

^a^Assessed in *n* = 23.

The median (range) of duration for treatment with nivolumab and mFOLFIRINOX regimen was 7.6 (0.1–31.1) months, and the follow-up duration was 13.4 (1.0–34.3) months. The mean (SD) relative dose intensity (%) was 98.7 (2.9), 76.8 (15.2), 85.0 (10.8), 71.4 (15.4) and 81.6 (12.9) for nivolumab, oxaliplatin, levofolinate, irinotecan and fluorouracil, respectively. Only one (3.2%) patient received <50% relative dose intensity of oxaliplatin while two (6.5%) patients received <50% relative dose intensity of irinotecan. The majority (*n* = 5, 80.6%) of patients discontinued as they had progressive disease as assessed by the investigator or sub-investigator according to RECIST v1.1. Data were available for subsequent anticancer therapy in 28 patients; of these, 19 (67.8%) received gemcitabine + nab-paclitaxel, four (14.3%) received gemcitabine, one (3.6%) received S-1 and one (3.6%) underwent a surgery, while three (10.7%) did not receive subsequent anticancer therapy.

### Efficacy

ORR (90% CI) by central assessment was 32.3% (18.7%–48.5%; Table 2) and the median duration of response was 7.4 (range: 3.5–21.9) months. The primary endpoint was not met as the lower limit of the 90% CI for the ORR was lower than the threshold ORR of 31.6%. ORR by PD-L1 status was 30.4% at CPS <1 and 42.9% at CPS ≥1 (Supplementary Table 1). ORR by TMB analysis showed that 45.0% (9/20) patients with TMB <5 achieved objective response while 0.0% (0/4) patients with TMB ≥5 achieved objective response (Supplementary Table 1).

**Table 2:** Best overall response by central assessment (total study population, *N* = 31).

Outcome	No. of patients with response	Rate, % (90% CI)
Objective response^a^	10	32.3 (18.7–48.5)
Complete response^b^	0	0 (0–9.2)
Partial response^c^	10	32.3 (18.7–48.5)
Stable disease	12	38.7 (24.1–55.0)
Progressive disease^d^	7	22.6
Not evaluable	2	6.5

^a^Comprises patients whose best overall response was complete or partial.

^b^Defined as disappearance of all non-nodal target lesions. Any nodal lesions must have reduction in short axis to <10 mm.

^c^Defined as at least a 30% decrease from baseline in the sum of diameters of target lesions.

^d^Defined as at least a 20% increase in the sum of diameters of target lesions relative to the smallest sum observed in the study and with an absolute increase of ≥5 mm in the sum.

*CI* confidence interval.

**Fig. 1:**
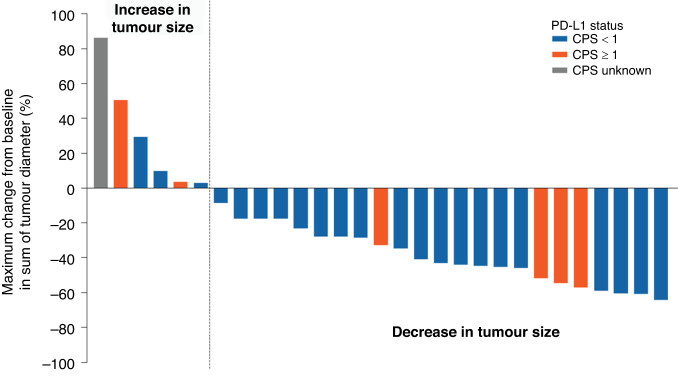
Maximum percentage change in tumour diameter from baseline. *CPS* combined positive score, *PD-L1* programmed death-ligand 1. Represented here are 29 evaluable patients with target lesion at baseline and at least one follow-up after the first administration of study treatment.

Maximum percentage change in tumour diameter from baseline showed an increase in six patients and a reduction in 23 patients (Fig. 1). Among patients with CPS <1, 86.4% (19/22) showed a decrease in tumour diameter, while among those with CPS ≥1, 66.7% (4/6) showed a decrease in tumour diameter.

**Fig. 2:**
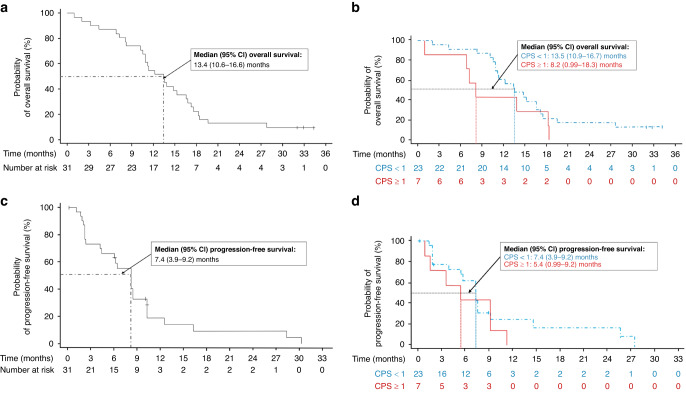
Survival plots. **a** Overall survival in the study population, **b** Overall survival by PD-L1 status, **c** Progression-free survival in the study population, **d** Progression-free survival by PD-L1 status. Analysis set: Full analysis set. 1 month = 30.4375 days. *CI* confidence interval, *CPS* combined positive score, *PDL-1* programmed death-ligand 1. The analysis by PD-L1 status was done for 30 patients whose CPS data were available. The 95% CIs were estimated using the Kaplan–Meier method.

The 1-year survival rate was 54.8% (95% CI: 36.0%–70.3%) (Supplementary Table 2), and the median OS was 13.4 (95% CI: 10.6–16.6) months in the study population (Fig. 2a). Median OS was 13.5 months in PD-L1 CPS <1 and 8.2 months in PD-L1 CPS ≥1 subgroups (Fig. 2b). The 6-month PFS rate was 55.5% (95% CI: 35.8%–71.3%) (Supplementary Table 2), and the median PFS was 7.4 (95% CI: 3.9–9.2) months in the study population (Fig. 2c). Median PFS was 7.4 months in PD-L1 CPS <1 and 5.4 months in PD-L1 CPS ≥1 subgroups (Fig. 2d). The OS and PFS rates at 6, 12, 18 and 24 months are presented in Supplementary Table 2.

### Safety

Major reason for discontinuation of nivolumab was progressive disease as assessed by the investigator or sub-investigator according to RECIST v1.1 (*n* = 25, 80.6%). Drug-related serious AEs were reported in 29.0% of the patients; 3.2% drug-related AEs led to discontinuation, and none led to death within 30-day safety window (Supplementary Table 3). Nausea (80.6%), diarrhoea (61.3%), neutrophil count decreased (61.3%) and peripheral sensory neuropathy (61.3%) were the most common AEs within 30-day safety window (Table 3). Diarrhoea (22.6%), rash (12.9%) and hypothyroidism (12.9%) were the most common treatment-emergent AEs of interest for nivolumab within 100-day safety window (Table 4).

**Table 3:** Summary of adverse events by preferred terms (30-day safety window).

Adverse event preferred term	Any grade	Grade 3/4
Nausea	25 (80.6)	3 (9.7)
Diarrhoea	19 (61.3)	1 (3.2)
Neutrophil count decreased	19 (61.3)	12 (38.7)
Peripheral sensory neuropathy	19 (61.3)	0
Decreased appetite	18 (58.1)	5 (16.1)
Stomatitis	15 (48.4)	0
Constipation	13 (41.9)	0
Dysgeusia	12 (38.7)	0
Malaise	14 (45.2)	1 (3.2)
Rash	10 (32.3)	1 (3.2)
White blood cell count decreased	9 (29.0)	3 (9.7)
Platelet count decreased	8 (25.8)	0
Alopecia	7 (22.6)	0
Fatigue	7 (22.6)	0
Hiccups	7 (22.6)	0
Pyrexia	7 (22.6)	0

Data are presented as *n* (%) for adverse events observed in ≥20% of the patients between the first day of the regimen and 30 days after the last dose.

There were no Grade 5 adverse events.

**Table 4:** Treatment-emergent adverse events of interest for nivolumab (100-day safety window).

Adverse events preferred term	Any grade	Grade 3/4
Diarrhoea	7 (22.6)	0
Rash	4 (12.9)	0
Hypothyroidism	4 (12.9)	0
Interstitial lung disease^a^	2 (6.5)	1 (3.2)
Gamma-glutamyltransferase increased	2 (6.5)	0
Alanine aminotransferase increased	1 (3.2)	0
Aspartate aminotransferase increased	1 (3.2)	0
Blood alkaline phosphatase increased	1 (3.2)	0
Adrenal disorder	1 (3.2)	0

Data are presented as *n* (%) for adverse events observed between the first day of the regimen and 100 days after the last dose.

There were no Grade 5 adverse events.

^a^Both events were serious drug-related adverse events.

## Discussion

The abysmally poor prognosis in advanced pancreatic cancer continues to drive the research for therapeutic options with better efficacy and/or safety. We assessed the efficacy and safety of nivolumab in combination with mFOLFIRINOX in 31 Japanese patients with chemotherapy-naïve pancreatic cancer with distant metastasis over a median follow-up of 13.4 months. The primary efficacy endpoint was not met; ORR was 32.3% (complete response 0.0%; partial response 32.3%). Median OS, however, was longer than a year (13.4 [95% CI: 10.6–16.6] months) with a 1-year survival of 54.8% (95% CI: 36.0%–70.3%), a median PFS of 7.4 (95% CI: 3.9–9.2) months, and a median duration of response of 7.4 (range: 3.5–21.9) months. The safety profile demonstrated that this combination was tolerable with no new safety signals.

Immunotherapy in combination with chemotherapy for pancreatic cancer is expected to improve efficacy, similar to that observed in other solid tumours [23, 31]. However, the combined efficacy of mFOLFIRINOX with nivolumab has not been assessed previously.

In our study, the median OS was 13.4 months and 54.8% individuals survived longer than a year. Median PFS was 7.4 months. The median OS and PFS in our study were numerically though not statistically better than the median OS and PFS reported in previous Phase 2 studies in Japanese patients with untreated metastatic pancreatic cancer (excluding patients with peritoneal dissemination): 10.7 and 5.6 months, respectively, with FOLFIRINOX treatment [32] and 11.2 and 5.5 months, respectively, with the mFOLFIRINOX regimen [33]. The safety profile in our study was favourable compared to these previous studies. Nevertheless, the ORR observed in the present study was comparable to that reported in previous studies [32, 33]. Further studies with larger sample sizes are needed to evaluate the efficacy and safety results of the combination with immune checkpoint inhibitor.

Similarly, comparisons can be drawn between our study with the previous open-label, Phase 1 study of nivolumab + nab-paclitaxel + gemcitabine in 50 patients with locally advanced/metastatic pancreatic cancer [34]. The baseline characteristics in that study were comparable to those in our study, except for some differences in ECOG performance status (0, 38% vs. 68%; 1, 62% vs. 32%) and probably for PD-L1 status (<1%: 56% vs. 74%; ≥1%, 24% vs. 23%; missing, 20% vs. 3%). With a median OS of 9.9 months (95% CI: 6.74–12.16 months) and a median PFS of 5.5 months (95% CI: 3.3–7.2 months), the authors concluded that the combination therapy with nivolumab did not lead to survival benefit. Though the OS and PFS in our study were numerically better than those reported by Wainberg et al. [34], our conclusion of no statistically significant efficacy benefit was similar. Also, in both trials, PD-L1 positivity was not associated with substantially better efficacy and the relative dose intensity was comparable. Further studies with larger sample sizes are warranted.

The use of valid biomarkers for individualised targeted treatment selection could improve outcomes [31]. Although we also evaluated the potential effects of PD-L1 expression and TMB on efficacy, there was no correlation between either PD-L1 expression or TMB and efficacy. Those with CPS ≥1 had lower numerical values for OS, PFS and the proportion demonstrating a decrease in tumour diameter, though they had higher ORR than those with CPS <1. A meta-analysis evaluating the effect of PD-L1 positive status on the prognosis of patients with pancreatic ductal adenocarcinoma demonstrated that high PD-L1 expression levels were associated with a poor prognosis [35]. Thus, even though this combination showed higher ORR in PD-L1 positive patients, it was not sufficient to prolong the OS. In terms of TMB, although high TMB values potentially predict response to immunotherapy [36], ORR was 0.0% at TMB ≥5 vs. 45.0% at TMB <5. It must be clarified that no patients having 10 or more mut/mb were observed amongst patients enroled in this study, thus the cutoff of 5 mut/mb was used for TMB instead of the commonly used 10 mut/mb cutoff. Overall, the results must be interpreted with caution given the small number of patients in the study and unequal distribution across subgroups.

Some limitations of this study should be discussed. This was a single-arm study limiting comparison with other treatment regimens, except from historical cohorts. Considering the small cohort size, we could not compare responders vs. non-responders to identify factors associated with combination therapy response. Also, the small sample size may have led to unreliable results for subgroup analyses based on biomarkers. Nevertheless, this study provides initial encouraging results of prolonged survival with nivolumab plus mFOLFIRINOX immunochemotherapy in advanced pancreatic carcinoma.

## Conclusions

Our results demonstrated that nivolumab in combination with modified FOLFIRINOX had a manageable safety profile in patients with metastatic pancreatic cancer. The primary endpoint for ORR was not met and the study failed to show meaningful increase in efficacy with nivolumab added to mFOLFIRINOX. Nevertheless, the ORR observed in this study was comparable to previously reported data. We found no correlation between PD-L1 expression and efficacy. In the late stages of pancreatic cancer, single immunotherapy with chemotherapy may have limited benefit and multiple immunotherapeutic options in combination with chemotherapy may improve efficacy. Further research is needed to determine the patient subpopulations which can benefit from this combination therapy.

## Supplementary information

Supplementary material

## Data Availability

Qualified researchers may request Ono Pharma to disclose individual patient-level data from clinical studies through the following website: https://www.clinicalstudydatarequest.com/. For more information on Ono Pharma’s Policy for the disclosure of clinical study data, please see the following website: https://www.ono.co.jp/eng/rd/policy.html.

## References

[1] Arnold M, Abnet CC, Neale RE, Vignat J, Giovannucci EL, McGlynn KA, et al. Global burden of 5 major types of gastrointestinal cancer. Gastroenterology. 2020;159:335–49. 32247694 10.1053/j.gastro.2020.02.068 PMC8630546

[2] Schawkat K, Manning MA, Glickman JN, Mortele KJ. Pancreatic ductal adenocarcinoma and its variants: pearls and perils. Radiographics. 2020;40:1219–39. 32678699 10.1148/rg.2020190184

[3] Xiao AY, Tan ML, Wu LM, Asrani VM, Windsor JA, Yadav D, et al. Global incidence and mortality of pancreatic diseases: a systematic review, meta-analysis, and meta-regression of population-based cohort studies. Lancet Gastroenterol Hepatol. 2016;1:45–55. 28404111 10.1016/S2468-1253(16)30004-8

[4] Ferlay J, Partensky C, Bray F. More deaths from pancreatic cancer than breast cancer in the EU by 2017. Acta Oncol. 2016;55:1158–60. 27551890 10.1080/0284186X.2016.1197419

[5] Rahib L, Smith BD, Aizenberg R, Rosenzweig AB, Fleshman JM, Matrisian LM. Projecting cancer incidence and deaths to 2030: the unexpected burden of thyroid, liver, and pancreas cancers in the United States. Cancer Res. 2014;74:2913–21. 10.1158/0008-5472.CAN-14-0155. 24840647 10.1158/0008-5472.CAN-14-0155

[6] NCCN Guidelines Version 2.2022 Pancreatic Adenocarcinoma https://www.nccn.org/professionals/physician_gls/pdf/pancreatic.pdf Last accessed: March 31, 2023. (2022).

[7] Okusaka T, Nakamura M, Yoshida M, Kitano M, Uesaka K, Ito Y, et al. Committee for Revision of Clinical Guidelines for Pancreatic Cancer of the Japan Pancreas Society. Clinical Practice Guidelines for Pancreatic Cancer 2019 from the Japan Pancreas Society: a synopsis. Pancreas. 2020;49:326–35. 32132516 10.1097/MPA.0000000000001513 PMC7077959

[8] Sohal DPS, Kennedy EB, Cinar P, Conroy T, Copur MS, Crane CH, et al. Metastatic pancreatic cancer: ASCO Guideline Update. J Clin Oncol. 2020;38:3217–30. 10.1200/JCO.20.01364. 10.1200/JCO.20.01364 PMC12974607 32755482

[9] Terashima T, Yamashita T, Sakai A, Ohta H, Hinoue Y, Toya D, et al. Treatment patterns and outcomes of unresectable pancreatic cancer patients in real-life practice: a region-wide analysis. Jpn J Clin Oncol. 2018;48:966–73. 30256958 10.1093/jjco/hyy132

[10] Singh RR, O’Reilly EM. New treatment strategies for metastatic pancreatic ductal adenocarcinoma. Drugs. 2020;80:647–69. 32306207 10.1007/s40265-020-01304-0 PMC7466866

[11] Klein-Brill A, Amar-Farkash S, Lawrence G, Collisson EA, Aran D. Comparison of FOLFIRINOX vs gemcitabine plus nab-paclitaxel as first-line chemotherapy for metastatic pancreatic ductal adenocarcinoma. JAMA Netw Open. 2022;5:e2216199. 35675073 10.1001/jamanetworkopen.2022.16199 PMC9178436

[12] Fan JQ, Wang MF, Chen HL, Shang D, Das JK, Song J. Current advances and outlooks in immunotherapy for pancreatic ductal adenocarcinoma. Mol Cancer. 2020;19:32. 32061257 10.1186/s12943-020-01151-3 PMC7023714

[13] Wainberg ZA, Melisi D, Macarulla T, Pazo-Cid R, Chandana SR, De La Fouchardiere C, et al. NAPOLI-3: A randomized, open-label phase 3 study of liposomal irinotecan + 5-fluorouracil/leucovorin + oxaliplatin (NALIRIFOX) versus nab-paclitaxel + gemcitabine in treatment-naïve patients with metastatic pancreatic ductal adenocarcinoma (mPDAC). J Clin Oncol. 2023;41:LBA661-LBA661. 10.1016/S0140-6736(23)01366-1 PMC11664154 37708904

[14] Wang C, Thudium KB, Han M, Wang XT, Huang H, Feingersh D, et al. In vitro characterization of the anti-PD-1 antibody nivolumab, BMS-936558, and in vivo toxicology in non-human primates. Cancer Immunol Res. 2014;2:846–56. 24872026 10.1158/2326-6066.CIR-14-0040

[15] Yang Y, Jin G, Pang Y, Huang Y, Wang W, Zhang H, et al. Comparative efficacy and safety of nivolumab and nivolumab plus ipilimumab in advanced cancer: a systematic review and meta-analysis. Front Pharmacol. 2020;11:40. 32116716 10.3389/fphar.2020.00040 PMC7033417

[16] Fizazi K, Mella PG, Castellano D, Minatta JN, Kalebasty AR, Shaffer D, et al. Nivolumab plus docetaxel in patients with chemotherapy-naïve metastatic castration-resistant prostate cancer: results from the phase II CheckMate 9KD trial. Eur J Cancer. 2022;160:61–71. 34802864 10.1016/j.ejca.2021.09.043

[17] Tie Y, Ma X, Zhu C, Mao Y, Shen K, Wei X, et al. Safety and efficacy of nivolumab in the treatment of cancers: a meta-analysis of 27 prospective clinical trials. Int J Cancer. 2017;140:948–58. 27813059 10.1002/ijc.30501

[18] Miller AL, Garcia PL, Yoon KJ. Developing effective combination therapy for pancreatic cancer: an overview. Pharmacol Res. 2020;155:104740. 32135247 10.1016/j.phrs.2020.104740 PMC7365261

[19] O’Hara MH, O’Reilly EM, Varadhachary G, Wolff RA, Wainberg ZA, Ko AH, et al. CD40 agonistic monoclonal antibody APX005M (sotigalimab) and chemotherapy, with or without nivolumab, for the treatment of metastatic pancreatic adenocarcinoma: an open-label, multicentre, phase 1b study. Lancet Oncol. 2021;22:118–31. 33387490 10.1016/S1470-2045(20)30532-5

[20] Chen DS, Mellman I. Oncology meets immunology: the cancer-immunity cycle. Immunity. 2013;39:1–10. 23890059 10.1016/j.immuni.2013.07.012

[21] Chen Y, Liu R, Li C, Song Y, Liu G, Huang Q, et al. Nab-paclitaxel promotes the cancer-immunity cycle as a potential immunomodulator. Am J Cancer Res. 2021;11:3445–60. 34354854 PMC8332864

[22] Zhu Y, Liu N, Xiong SD, Zheng YJ, Chu YW. CD4+Foxp3+ regulatory T-cell impairment by paclitaxel is independent of toll-like receptor 4. Scand J Immunol. 2011;73:301–8. 21223350 10.1111/j.1365-3083.2011.02514.x

[23] Zhang J, Pan S, Jian C, Hao L, Dong J, Sun Q, et al. Immunostimulatory properties of chemotherapy in breast cancer: from immunogenic modulation mechanisms to clinical practice. Front Immunol. 2022;12:819405. 35069604 10.3389/fimmu.2021.819405 PMC8766762

[24] Shurin GV, Tourkova IL, Kaneno R, Shurin MR. Chemotherapeutic agents in noncytotoxic concentrations increase antigen presentation by dendritic cells via an IL-12-dependent mechanism. J Immunol. 2009;183:137–44. 19535620 10.4049/jimmunol.0900734 PMC4005417

[25] Ma Y, Adjemian S, Mattarollo SR, Yamazaki T, Aymeric L, Yang H, et al. Anticancer chemotherapy-induced intratumoral recruitment and differentiation of antigen-presenting cells. Immunity. 2013;38:729–41. 23562161 10.1016/j.immuni.2013.03.003

[26] Apetoh L, Végran F, Ladoire S, Ghiringhelli F. Restoration of antitumor immunity through selective inhibition of myeloid derived suppressor cells by anticancer therapies. Curr Mol Med. 2011;11:365–72. 21568934 10.2174/156652411795976574

[27] Ghiringhelli F, Apetoh L. Enhancing the anticancer effects of 5-fluorouracil: current challenges and future perspectives. Biomed J. 2015;38:111–6. 25163503 10.4103/2319-4170.130923

[28] Zitvogel L, Galluzzi L, Smyth MJ, Kroemer G. Mechanism of action of conventional and targeted anticancer therapies: reinstating immunosurveillance. Immunity. 2013;39:74–88. 23890065 10.1016/j.immuni.2013.06.014

[29] Oken MM, Creech RH, Tormey DC, Horton J, Davis TE, McFadden ET, et al. Toxicity and response criteria of the Eastern Cooperative Oncology Group. Am J Clin Oncol. 1982;5:649–55. 7165009

[30] Conroy T, Desseigne F, Ychou M, Bouché O, Guimbaud R, Bécouarn Y, et al. FOLFIRINOX versus gemcitabine for metastatic pancreatic cancer. N Engl J Med. 2011;364:1817–25. 21561347 10.1056/NEJMoa1011923

[31] Schizas D, Charalampakis N, Kole C, Economopoulou P, Koustas E, Gkotsis E, et al. Immunotherapy for pancreatic cancer: a 2020 update. Cancer Treat Rev. 2020;86:102016. 32247999 10.1016/j.ctrv.2020.102016

[32] Okusaka T, Ikeda M, Fukutomi A, Ioka T, Furuse J, Ohkawa S, et al. Phase II study of FOLFIRINOX for chemotherapy-naïve Japanese patients with metastatic pancreatic cancer. Cancer Sci. 2014;105:1321–6. 25117729 10.1111/cas.12501 PMC4462360

[33] Ozaka M, Ishii H, Sato T, Ueno M, Ikeda M, Uesugi K, et al. A phase II study of modified FOLFIRINOX for chemotherapy-naïve patients with metastatic pancreatic cancer. Cancer Chemother Pharmacol. 2018;81:1017–23. 29633005 10.1007/s00280-018-3577-9

[34] Wainberg ZA, Hochster HS, Kim EJ, George B, Kaylan A, Chiorean EG, et al. Open-label, phase I study of nivolumab combined with nab-paclitaxel plus gemcitabine in advanced pancreatic cancer. Clin Cancer Res. 2020;26:4814–22. 32554514 10.1158/1078-0432.CCR-20-0099

[35] Gao HL, Liu L, Qi ZH, Xu HX, Wang WQ, Wu CT, et al. The clinicopathological and prognostic significance of PD-L1 expression in pancreatic cancer: a meta-analysis. Hepatobiliary Pancreat Dis Int. 2018;17:95–100. 29576277 10.1016/j.hbpd.2018.03.007

[36] Lawlor RT, Mattiolo P, Mafficini A, Hong SM, Piredda ML, Taormina SV, et al. Tumor mutational burden as a potential biomarker for immunotherapy in pancreatic cancer: systematic review and still-open questions. Cancers (Basel). 2021;13:3119. 34206554 10.3390/cancers13133119 PMC8269341

