# Supplementary material for: A Phase 2 study of nivolumab in combination with modified FOLFIRINOX for metastatic pancreatic cancer
Source: BJC Rep. 2024 Jan 23;2:3. doi: 10.1038/s44276-023-00028-4 (PMC11524122; doi:10.1038/s44276-023-00028-4)
Supplement: Supplementary file 1 — Supplementary material [file 44276_2023_28_MOESM1_ESM.docx]

**Supplementary material:**

**Autoimmune diseases**:

Patients who had any chronic or recurrent autoimmune disease listed below at the time of or before enrolment were not enrolled in this clinical trial. For a patient with any other autoimmune disease, the investigators carefully assessed the eligibility of the patient and if there any were safety concerns, such patient was not enrolled in this trial.

- Acute disseminated encephalomyelitis
- IgA nephropathy
- Addison’s disease
- Inflammatory bowel disease
- Alopecia universalis
- Interstitial cystitis
- Ankylosing spondylitis
- Lambert-Eaton myasthenic syndrome
- Antiphospholipid syndrome
- Lupus erythematosus
- Aplastic anemia
- Lyme disease (chronic)
- Asthma
- Meniere’s syndrome
- Autoimmune hemolytic anemia
- Mooren’s ulcer
- Autoimmune hepatitis
- Morphea
- Autoimmune hypophysitis
- Multiple sclerosis
- Autoimmune hypoparathyroidism
- Myasthenia gravis
- Autoimmune myocarditis
- Neuromyotonia
- Autoimmune oophoritis
- Opsoclonus myoclonus syndrome
- Autoimmune orchitis
- Optic neuritis
- Autoimmune thrombocytopenic purpura
- Ord’s thyroiditis
- Behçet’s disease
- Pemphigus
- Bullous pemphigoid
- Pernicious anemia
- Celiac disease
- Polyarteritis nodosa
- Chronic fatigue syndrome
- Polyarthritis
- Chronic inflammatory demyelinating polyradiculopathy
- Autoimmune polyglandular syndrome
- Churg-Strauss syndrome
- Primary biliary cirrhosis
- Crohn’s disease (gastrointestinal ulceration)
- Psoriasis
- Dermatomyositis
- Reiter’s syndrome
- Type 1 diabetes mellitus
- Rheumatoid arthritis
- Dysautonomia
- Sarcoidosis
- Eczema
- Scleroderma
- Sjögren’s syndrome
- Acquired epidermolysis bullosa
- Stiff-man syndrome
- Pemphigoid gestationis
- Takayasu’s arteritis
- Giant cell arteritis
- Ulcerative colitis
- Goodpasture’s syndrome
- Graves’ disease
- Vogt-Koyanagi-Harada syndrome
- Guillain-Barre syndrome
- Vulvodynia
- Hashimoto’s disease
- Wegener’s granulomatosis
- Kawasaki disease

**Cumulative dose intensity calculation:**

Cumulative dose of each drug in mFOLFIRINOX regimen (mg/m^2^) was the sum of dose level (mg/m^2^) administered to a subject during the treatment period. If the value of dose was unknown, it was considered as "0" for the dose calculation.

Relative dose intensity of each drug in mFFX regimen (%) was calculated by the following equation.

Oxaliplatin:

Relative dose intensity (%) =$\frac{Cumulative dose (mg/m^{2})}{\frac{Date of the last dose - Date of the first dose + 14 \left[ \mathrm{days} \right]}{28 (days)} x 85 mg/m^{2}x 2}x 100$

Levofolinate;

Relative dose intensity (%) =$\frac{Cumulative dose (mg/m^{2})}{\frac{Date of the last dose - Date of the first dose + 14 (days)}{28 (days)} x 200 mg/m^{2} x 2}x 100$

Irinotecan;

Relative dose intensity (%) =$\frac{Cumulative dose (mg/m^{2})}{\frac{Date of the last dose - Date of the first dose + 14 (days)}{28 (days)} x 150 mg/m^{2} x 2}x 100$

Fluorouracil;

Relative dose intensity (%) =$\frac{Cumulative dose (mg/m^{2})}{\frac{Date of the last dose - Date of the first dose + 14 (days)}{28 (days)} x 2400 mg/m^{2} x 2}x 100$

**Supplementary Table 1.** Objective response rate (by central assessment).

| **Outcome** | **No. of patients with objective response** | **Objective response rate,^a^ % (90% CI)** |
| --- | --- | --- |
| ***By PD-L1 status in tumour cells*** | | |
| CPS <1 (*n* = 23) | 7 | 30.4 (15.2–49.6) |
| CPS ≥1 (*n* = 7) | 3 | 42.9 (12.9–77.5) |
| ***By TMB*** | | |
| TMB <5 (*n* = 20) | 9 | 45.0 (25.9–65.3) |
| TMB ≥5 (*n* = 4) | 0 | 0.0 (0.0–52.7) |

*CPS* combined positive score, *n* number of patients in each category, *PD-L1* programmed death-ligand 1, *TMB* tumour mutation burden.

^a^Includes patients whose best overall response was complete or partial.

**Supplementary Table 2.** OS and PFS at different timepoints

|  | **OS [95% CI]** | **PFS [95% CI]** |
| --- | --- | --- |
| Month 6 | 87.1 [69.2–95.0] | 55.5 [35.8–71.3] |
| Month 12 | 54.8 [36.0–70.3] | 14.6 [4.0–31.6] |
| Month 18 | 22.6 [10.0–38.3] | 9.7 [1.8–25.9] |
| Month 24 | 12.9 [4.1–27.0] | 9.7 [1.8–25.9] |

*OS* overall survival; *PFS* progression-free survival

**Supplementary Table 3.** Summary of adverse events and drug-related adverse events (30-day safety window)

| **Adverse events** | **Any Grade** | **Grade 3/4** |
| --- | --- | --- |
| Number of patients with AEs | 31 (100.0) | 25 (80.6) |
| Number of patients with SAEs | 15 (48.4) | 13 (41.9) |
| Number of patients with AEs leading to discontinuation of nivolumab or mFOLFIRINOX regimen | 1 (3.2) | 1 (3.2) |
| Number of patients with AEs leading to drug interruption of nivolumab or mFOLFIRINOX regimen | 29 (93.5) | 18 (58.1) |
| Number of patients with AEs leading to death | 0 | 0 |
| Number of patients with drug-related AEs | 31 (100.0) | 20 (64.5) |
| Number of patients with drug-related SAEs | 9 (29.0) | 8 (25.8) |
| Number of patients with drug-related AEs leading to discontinuation of nivolumab or mFOLFIRINOX regimen | 1 (3.2) | 1 (3.2) |
| Number of patients with drug-related AEs leading to drug interruption of nivolumab or mFOLFIRINOX regimen | 28 (90.3) | 16 (51.6) |
| Number of patients with drug-related AEs leading to death | 0 | 0 |

CTCAE version 4.0.

*AEs* adverse events, *SAEs* serious adverse events.

There were no Grade 5 AEs.

Drug-related AEs occurring between the start date of the first administration of nivolumab and 30 days after the last dose of nivolumab or mFOLFIRINOX regimen were tabulated.

Drug-related AEs were defined as any AEs with causal relationship to nivolumab or mFOLFIRINOX regimen assessed as “Related” or missing.
